# Simulation of a Rohksar–Kivelson ladder on a NISQ device

**DOI:** 10.1038/s41598-024-79480-2

**Published:** 2024-11-26

**Authors:** Sabhyata Gupta, Younes Javanmard, Tobias J. Osborne, Luis Santos

**Affiliations:** 1 Institut für Theoretische Physik, Leibniz Universität Hannover, Appelstr. 2, 30167 Hannover, Germany; 2 PASQAL, 7 rue Léonard de Vinci, 91300 Massy, France

**Keywords:** Quantum simulation, Theoretical physics

## Abstract

We present a quantum-classical algorithm to study the dynamics of the Rohksar–Kivelson plaquette ladder on NISQ devices. We show that complexity is largely reduced using gauge invariance, additional symmetries, and a crucial property associated to how plaquettes are blocked against ring-exchange in the ladder geometry. This allows for an efficient simulation of sizable plaquette ladders with a small number of qubits, well suited for the capabilities of present NISQ devices. We illustrate the procedure for ladders with simulation of up to 8 plaquettes in an IBM-Q machine, employing scaled quantum gates.

**Supplementary Information:**

The online version contains supplementary material available at 10.1038/s41598-024-79480-2.

## Introduction

Recent years have witnessed spectacular progress in the field of quantum simulators^1,2^, i.e. quantum many-particle systems that simulate other quantum phenomena, which may not be tractable by classical means. This includes applications in disparate fields, ranging from condensed-matter physics^3,4^ to material science and quantum chemistry^5,6^ and high-energy physics^7,8^. Concerning the latter, quantum simulators open exciting possibilities for the simulation of quantum field theories, and in particular lattice gauge theories with both analogue and digital simulators^7–13^. A particularly prominent example is provided by the successful simulation of the quantum link Schwinger model^14^, a relatively simple model that describes quantum electrodynamics in one space and one time dimension^15–20^.

Despite these extraordinary developments, the analogue simulation of plaquette operators, crucial in the Hamiltonian formulation of lattice gauge theories^21^, remains very challenging, since they involve three- and higher-body interactions. Interestingly, it has been recently proposed that the Rydberg blockade in Rydberg configurable arrays could be employed to efficiently simulate plaquette terms, and in particular the Rohksar–Kivelson (RK) model, a two-dimensional U(1) lattice gauge theory which has attracted a large deal of interest due to its relevance in the context of quantum dimer and spin ice theory^22^.

Digital simulations may overcome the limitations of analogue devices concerning plaquette operators^23–25^. However, at the present time, fully fault-tolerant quantum computers are not available. Instead, only noisy intermediate-scale quantum (NISQ) devices^26^ have been so far realised, with at most hundreds of qubits, and characterized by sparse connectivity and significant noise and decoherence in the application of quantum gates. Since quantum error correction is not yet possible, a set of error mitigation techniques^27,28^, and algorithms^29^ have been specifically designed for NISQ devices. In particular different variational quantum algorithms have been explored for the simulation of the Schwinger model^17,30–33^.

**Fig. 1:**
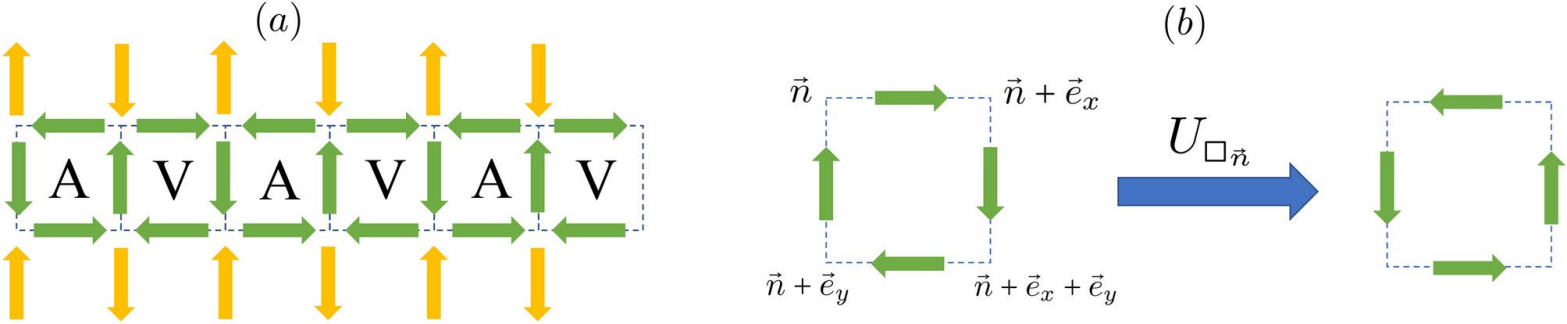
(**a**) Schematic representation of the RK ladder. Green arrows indicate the spin-1/2 particles associated to each link of the ladder. Note that for the horizontal links we employ the notation $\rightarrow$ ($\leftarrow$) to indicate spin $\uparrow$ ($\downarrow$). The orange arrows denote virtual links, which we employ to define the local Gauss law at each lattice vertex (see text). The green spins are placed in the AVAVAV configuration, which we consider as the initial state in our simulations. (**b**) Application of the ring-exchange operator $U_{\square _{\textbf{n}}}$ over the spins of plaquette $\square _{\textbf{n}}$.

Here, we apply a quantum-classical approach, inspired by the motivation behind that employed in Ref.^17^, for the study of the real-time dynamics of the RK model in a ladder geometry, possibly the simplest lattice gauge model with plaquette operators. We show that the combination of gauge invariance, symmetrization, and the particular way plaquettes are blocked against ring-exchange in the ladder geometry, allow for an efficient mapping of the RK-ladder dynamics to a small number of qubits, an interesting feature for its implementation in NISQ devices. By employing gate scaling techniques, we report that the procedure allows for the successful digital quantum simulation of the dynamics of up to 8 plaquette RK ladders on IBM-Q superconducting quantum device.

## Model and mapping

We consider a square ladder, as in Fig. 1a. We characterize each ladder vertex by a vector $\textbf{n}=(n_x,n_y)$, with $n_y=1,2$ denoting the lower and upper legs, respectively. We associate to each link of the ladder a spin-1/2 degree of freedom, with two states $|\uparrow \rangle$ and $|\downarrow \rangle$. For convenience of the representation, for spins placed in links along the legs we denote those states as $|\rightarrow \rangle$ and $|\leftarrow \rangle$, respectively. Introducing the raising and lowering spin operators $S_{\textbf{n},\textbf{n}'}^\pm$ acting on the spin placed at the link between the neighboring sites $\textbf{n}$ and $\textbf{n}'$ ($S_{\textbf{n}, \textbf{n}'}^+ |\downarrow \rangle = |\uparrow \rangle$, $S_{\textbf{n}, \textbf{n}'}^- |\uparrow \rangle = |\downarrow \rangle$), we define for each plaquette $\square _{\textbf{n}}$ (with $\textbf{n}$ the top-left vertex of the plaquette, see Fig. 1b) a ring-exchange operator

1 $$\begin{aligned} U_{\square _{\textbf{n}}}= & S_{\textbf{n}, \textbf{n} + \textbf{e}_x}^- S_{\textbf{n} + \textbf{e}_x, \textbf{n} + \textbf{e}_x + \textbf{e}_y}^+ S_{\textbf{n} + \textbf{e}_y, \textbf{n} + \textbf{e}_x + \textbf{e}_y}^+ S_{\textbf{n}, \textbf{n} + \textbf{e}_y}^-, \end{aligned}$$

with $\textbf{e}_x=(1,0)$ and $\textbf{e}_y=(0,1)$. Given a vortex (antivortex) plaquette state $|V\rangle \equiv |\rightarrow ,\downarrow ,\leftarrow ,\uparrow \rangle$ ($|A\rangle \equiv |\leftarrow ,\uparrow ,\rightarrow ,\downarrow \rangle$), where we follow the same clockwise order of Fig. 1b, the ring-exchange flips the state as $U_{\square _{\textbf{n}}}|V\rangle = |A\rangle$, and $U_{\square _{\textbf{n}}}^\dagger |A\rangle = |V\rangle$. Any other spin configuration, which we denote below as $|B\rangle$, is blocked, i.e. non-flippable, and $U_{\square _{\textbf{n}}}|B\rangle =0$. We consider the RK model^34^:

2 $$\begin{aligned} H = \sum _{\square _{\textbf{n}}} \left[ -J( U_{\square _{\textbf{n}}} + U_{\square _{\textbf{n}}}^\dagger ) + \lambda (U_{\square _{\textbf{n}}} + U_{\square _{\textbf{n}}}^\dagger )^2 \right] , \end{aligned}$$

where $\sum _{\square _{\textbf{n}}}$ denotes the sum over all ladder plaquettes. Since $U_{\square _{\textbf{n}}}^2|V\rangle = |V\rangle$, $U_{\square _{\textbf{n}}}^{\dagger 2}|A\rangle = |A\rangle$, $U_{\square _{\textbf{n}}}^2|B\rangle = 0$, then the operator ${{\hat{F}}} = \sum _{\square _{\textbf{n}}} (U_{\square _{\textbf{n}}} + U_{\square _{\textbf{n}}}^\dagger )^2$ acts as counter of the number of flippable, either $|V\rangle$ or $|A\rangle$, plaquettes. The RK coupling $\lambda$ hence determines the energy penalty resulting from the change in the number of flippable plaquettes. We set below $J=1$.

We define the local gauge transformation generator at vertex $\textbf{n}$ as:

3 $$\begin{aligned} {\hat{G}}_{\textbf{n}} = {\hat{S}}_{\textbf{n}, \textbf{n} + \textbf{e}_x}^z - {\hat{S}}_{\textbf{n} - \textbf{e}_x,\textbf{n}}^z +{\hat{S}}_{\textbf{n}, \textbf{n} + \textbf{e}_y}^z - {\hat{S}}_{\textbf{n} - \textbf{e}_y, \textbf{e}_n}^z, \end{aligned}$$

with ${\hat{S}}_{\textbf{n}, \textbf{n}'}^z$ the *z* projection of the spin placed in between two neighboring sites $\textbf{n}$ and $\textbf{n}'$. Note that in the ladder geometry, we have to consider virtual spins outside the ladder, which remain fixed during the dynamics (orange arrows in Fig. 1). As a result, the gauge generators become of the form

4 $$\begin{aligned} {\hat{G}}_{2n,1} & = {\hat{S}}_{(2n,1),(2n+1,1)}^z - {\hat{S}}_{(2n-1,1),(2n,1)}^z \nonumber \\ & \quad + {\hat{S}}_{(2n,1),(2n,2)}^z+1/2,\end{aligned}$$

5 $$\begin{aligned} {\hat{G}}_{2n+1,1} & = {\hat{S}}_{(2n+1,1),(2n+2,1)}^z -{\hat{S}}_{(2n,1),(2n+1,1)}^z \nonumber \\ & \quad + {\hat{S}}_{(2n+1,1),(2n+1,2)}^z-1/2, \end{aligned}$$

6 $$\begin{aligned} {\hat{G}}_{2n,2} & = {\hat{S}}_{(2n,2),(2n+1,2)}^z-{\hat{S}}_{(2n-1,2),(2n,2)}^z \nonumber \\ & \quad - {\hat{S}}_{(2n,1),(2n,2)}^z+1/2, \end{aligned}$$

7 $$\begin{aligned} {\hat{G}}_{2n+1,2} & = {\hat{S}}_{(2n+1,2),(2n+2,2)}^z -{\hat{S}}_{(2n,2),(2n+1,2)}^z \nonumber \\& \quad - {\hat{S}}_{(2n+1,1),(2n+1,2)}^z-1/2. \end{aligned}$$

The RK-ladder Hamiltonian is then gauge invariant, $[{\hat{H}}, {\hat{G}}_{\textbf{n}}]=0$, and thus the physical Hilbert space is also gauge invariant, i.e. it splits into sectors of eigenstates of $G_{\textbf{n}}$. In the following, we focus on the charge-free sector, i.e. on states $|\psi \rangle$ that fulfill $G_{\textbf{n}}|\psi \rangle = 0$, although a similar procedure as that discussed below can be applied to other sectors. We assume as well periodic boundary conditions along the ladder axis *x*.

### Mapping

**Fig. 2:**
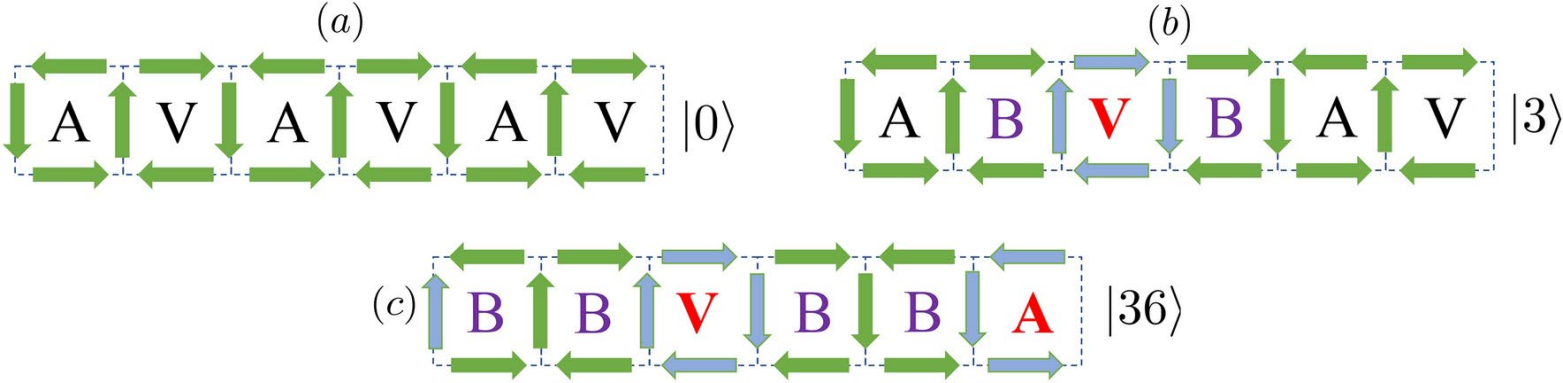
(**a**) Initial plaquette configuration, $|0\rangle$. (**b**) The third plaquette is flipped, resulting in the state $|3\rangle$. (**c**) The third and the sixth plaquettes are flipped, leading to the state $|3,6\rangle$. Note that we do not need to determine explicitly the blocked plaquettes, or follow why they are blocked. The states are fully defined by stating which plaquettes have been flipped compared to the initial state (**a**).

We are interested in the dynamics of the ladder plaquette, starting from a given initial configuration. A ladder with *N* plaquettes (and periodic boundary conditions) contains 3*N* spins, and hence presents in principle $2^{3N}$ spin configurations, which would hence naively require 3*N* qubits for its simulation. A more efficient procedure is possible, however, which we illustrate for the specific example of an RK-ladder with 6 plaquettes initially prepared in an AVAVAV configuration. We denote this initial state as $|0\rangle$, which has obviously 6 flippable plaquettes (see Fig. 2a). Other initial states are possible, which will modify the specific mapping discussed below, but similar mappings may be found as well.

Flipping a plaquette blocks the two neighboring ones (see Fig. 2b,c). For the mapping discussed below, it is crucial that in a ladder geometry, if flipping a plaquette blocks a neighboring one, the latter cannot be unblocked (i.e. transformed back into a flippable plaquette) by flipping a third one. Due to this key property, and if we are only interested in the flippable or non-flippable character of the plaquettes, we do not need to track why a plaquette is blocked. This is crucially different in a two-dimensional (2D) square lattice, where a blocked plaquette may be unblocked by flipping all the neighboring ones. As a result, in 2D geometries it is necessary to keep track why a plaquette is blocked, which results in a much more resource-consuming algorithm.

Due to the above-mentioned property, we may build the relevant states in a plaquette ladder by considering subsequent flips, without the need of describing the blocked plaquettes. We just need to keep track of which plaquettes have been flipped with respect to $|0\rangle$ (denoted in bold red in the examples of Fig. 2b,c), with the proviso that two neighboring plaquettes cannot be flipped. In the following, we employ the notation $|ijk\dots \rangle$, which denote ladders in which plaquettes $i,j,k,\dots$ have been flipped with respect to the initial state $|0\rangle$. Figure 2b,c show, respectively, the case of the states $|3\rangle$ and $|36\rangle$. Successive application of flips results in different families of states. States within a given family can be obtained from each other by translation taking into account periodic boundary conditions.

For the case of $N=6$ plaquettes, the states with only one flip, $|j\rangle$, $j=1,\dots 6$, have four remaining flippable plaquettes, see the example of Fig. 2b. Clearly these states form a single family. States with two flips split into two families: $\{ |13\rangle ,|15\rangle ,|24\rangle ,|26\rangle ,|35\rangle ,|46\rangle \}$, with states with three flippable plaquettes, and $\{|14\rangle ,|25\rangle ,|36\rangle \}$, which have two flippable plaquettes. Finally, there are states with three flips $\{|135\rangle , |246\rangle \}$, which have three flippable plaquettes each. Any other of the $2^{18}$ possible spin configurations of the ladder cannot be reached from $|0\rangle$, and hence does not need to be considered. The relevant Hilbert space fragment contains 18 states. However, the number of states taking part in the dynamics is significantly smaller, since flips only link $|\psi _0\rangle \equiv |0\rangle$ to the symmetric superpositions of the states of each family:

8 $$\begin{aligned} |\psi _{1}\rangle & = \frac{1}{\sqrt{6}} (|1\rangle +|2\rangle +|3\rangle +|4\rangle +|5\rangle +|6\rangle ),\end{aligned}$$

9 $$\begin{aligned} |\psi _{2}\rangle & = \frac{1}{\sqrt{6}} (|13\rangle +|15\rangle +|24\rangle +|26\rangle +|35\rangle +|46\rangle ), \end{aligned}$$

10 $$\begin{aligned} |\psi _{3}\rangle & = \frac{1}{\sqrt{3}} (|14\rangle +|25\rangle +|36\rangle ),\end{aligned}$$

11 $$\begin{aligned} |\psi _{4}\rangle & = \frac{1}{\sqrt{2}} (|135\rangle +|246\rangle ), \end{aligned}$$

which form a closed set of basis states linked by the effective Hamiltonian $\hat{\mathcal {H}}_{eff}=-\hat{\mathcal {H}}_{eff}^{(0)} + \lambda \hat{\mathcal {H}}_{eff}^{(1)}$, where

12 $$\begin{aligned} \hat{\mathcal {H}}_{eff}^{(0)} & = \sqrt{6}|\psi _0\rangle \langle \psi _1| + 2|\psi _1\rangle \langle \psi _2| \nonumber \\ & \quad + \sqrt{2} |\psi _1\rangle \langle \psi _3| + \sqrt{3} |\psi _3\rangle \langle \psi _4| +\mathrm {H.c.} \end{aligned}$$

13 $$\begin{aligned} \hat{\mathcal {H}}_{eff}^{(1)} & = 6|\psi _0\rangle \langle \psi _0| + 4|\psi _1\rangle \langle \psi _1|+ 3|\psi _2\rangle \langle \psi _2| \nonumber \\ & \quad + 2|\psi _3\rangle \langle \psi _3|+ 3|\psi _4\rangle \langle \psi _4|. \end{aligned}$$

Expanding the physical state in the state basis: $|\psi (t)\rangle =\sum _n c_n(t) |\psi _n\rangle$, we then obtain the equation for the time evolution of the amplitudes $c_n$: $i\hbar \dot{c}_n(t) = \sum _m \langle \psi _m|\hat{\mathcal {H}}_{eff}|\psi _n\rangle c_m(t)$. Note that the large reduction in complexity, from $2^{18}$ basis states to just five effective states for the case of 6-plaquette ladders, is based on the combination of gauge invariance, the above-mentioned property concerning blocked plaquettes, and symmetrization. This reduction of complexity allows for an efficient simulation of RK-ladders of a sizable number of plaquettes with a small number of qubits, well-suited for NISQ devices (see Methods).

The previous discussion has illustrated the procedure for a small number $N=6$ of plaquettes. Simulations of larger number of plaquettes demand the construction of the families of states with different number of flips that are equivalent when applying translation under periodic boundary conditions. This may be easily generated combinatorially using three constraints: the number of plaquettes flipped with respect to the initial configuration, the number of flippable plaquettes remaining, and the number of plaquettes between any flipped plaquettes in that state. After forming the families of states, each family contributes to one basis state $|\psi _n\rangle$, given by the symmetrization of all the states in the family. Once the basis states are determined, the coefficients of the effective Hamiltonian $\hat{\mathcal {H}}_{eff}$ are easily obtained from the normalization of the basis states. The preparation of the basis states and equations, crucial for the proposed algorithm, may be hence performed by means of a relatively simple classical numerical procedure. The implementation of the classical procedure can be found here^35^.

The number of effective states determines the number of equations $N_{EQ}$ to determine the time evolution, which demands the use of $Q=\log _2N_{EQ}$ qubits. For larger *N*, $Q\simeq 0.6N$, hence reducing the number of necessary qubits by a factor of $\simeq 5$ compared to the naive mapping of all spin states. For example, a sizable RK-ladder with 17 plaquettes (211 equations) can be simulated, if noise is properly harnessed, with just 8 qubits.

## Results

In order to evaluate the dynamics and benchmark the results obtained using a quantum device, we monitor two observables that characterize the dynamics in the RK ladder: the average number of flippable plaquettes $\langle {\hat{F}} \rangle$, and the plaquette-plaquette correlations

14 $$\begin{aligned} \mathcal {C}_{r} = \left\langle \left( U_{\square _{\textbf{n}}} + U_{\square _{\textbf{n}}}^\dagger \right) ^2 \left( U_{\square _{\textbf{n} + r\textbf{e}_x}} + U_{\square _{\textbf{n} + r\textbf{e}_x}}^\dagger \right) ^2\right\rangle . \end{aligned}$$

Note that due to periodic boundary conditions, the correlation is independent of the particular choice of $\textbf{n}$. The quantum simulation results discussed below were obtained using the noise model of the 7-qubit IBM-Q Lagos ($ibmq_lagos$ v 0.33). The noise model is openly available within IBM Quantum documentation. In each simulation, we execute the quantum circuit for the unitary time evolution operator for Trotter steps with a given time step $\delta t$ and measure counts with 8192 shots per measurement.

Figure 4a shows our results for $\lambda =1$ and different number *N* of plaquettes of $\langle \hat{F}\rangle (t)$ up to a time $t=10/J$. The figure compares our results using exact-diagonalization (ED, employing Krylov subspace), the ideal simulator, and the noisy circuit with non-scaled and scaled gates.

**Fig. 3:**
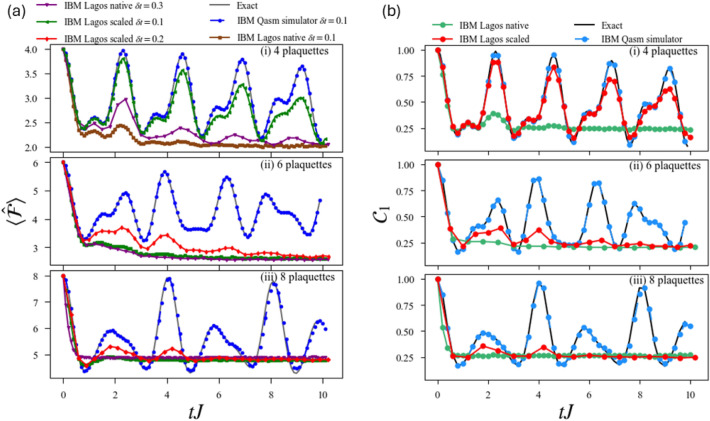
(**a**) Average number of flippable plaquettes $\langle {\hat{F}} \rangle$ as a function of time for an RK-ladder with $\lambda =1$ and  (i) 4  (ii) 6  (iii) 8 plaquettes. We compare the results obtained from exact time evolution, the ideal simulator, and the noisy circuit with non-scaled and scaled gates for different Trotter steps $\delta t$ (see legend over Fig. (a)). (**b**) Plaquette-plaquette correlation $\mathcal {C}_{1}$ as a function of time for an RK-ladder with $\lambda =1$ and  (i) 4  (ii) 6  (iii) 8 plaquettes. We compare the results obtained from exact time evolution, the ideal simulator, and the noisy circuit with non-scaled and scaled gates for Trotter step $\delta t= 0.3$ for non-scaled and $\delta t= 0.1$ for scaled gates.

The minimal case of $N=2$ plaquettes can be solved with a single qubit, and as a result the two-plaquette quantum simulation in a noisy device matches exceptionally well with the ED calculations and ideal simulator calculations (not shown). The advantage of using scaled quantum gates becomes evident when considering the case of $N=4$ plaquettes. The simulation for a noisy device with scaled gates with a Trotter step $\delta t= 0.1$ is in very good agreement with the ideal simulation, giving significant improvement over the noisy quantum simulation with the native gates as seen in Fig. 3a(i), which clearly fails already at $t=0.5J$.

For $N=6$ plaquettes, the number of CNOT gates is so large in the basis circuits that noisy quantum simulation with basis gates provides no useful data already for times $t > 0.7J$. In contrast, we see from Fig. 3a(ii) that the quantum simulation using scaled gates for a Trotter step $\delta t= 0.2$ recovers the qualitative behaviour of the ideal quantum simulation, which matches well with the ED calculation. For $N=8$ plaquettes the Trotter error remains very small in the ideal simulation. Again the noisy basis circuit simulation fails to recover useful data for any $t>0.2/J$, whereas the scaled gate simulation for a time step $\delta t= 0.2$ recovers the qualitative oscillation up to $t= 5J$, see Fig. 3a(iii).

Figure 3b shows our results for the nearest neighbor correlation $C_{1}$ for RK-ladders with $N=4$, 6 and 8 plaquettes. We compared again the ED results, the ideal quantum simulation, and the noisy quantum simulation with non-scaled and scaled gates, using $\delta t=0.3$ for non-scaled gates and $\delta t=0.1$ for scaled gates. These results show again that for $N=4$ noisy quantum simulations with re-scaled gates result in an excellent quantitative agreement with ED calculations, whereas those with basis gates fail already at short times $t>2/J$. Moreover, we observe again that calculations with re-scaled gates provide a qualitatively correct behavior for $t<5/J$ for $N=6$ and $N=8$, whereas the calculations with basis gates produce useless results already for $t>0.2/J$.

## Discussion

We have shown that the dynamics of square Rohksar–Kivelson ladders, a basic lattice gauge model, can be efficiently mapped onto a small number of qubits due to the combination of gauge invariance, symmetrization, and the particular way plaquettes are blocked against ring-exchange. These properties lead to a scalable mapping and reduce by a factor of 5 the number of necessary qubits to simulate the dynamics of the square RK ladder, an interesting feature for simulations in NISQ devices (for triangular Rohksar–Kivelson ladders^25^ the reduction is by a factor 3.3). We have illustrated the procedure using the simulator (including noise) of the 7-qubit IBM-Q machine Lagos, showing that the use of scaled gates allows for the faithful simulation of ladders of $N=4$ plaquettes. Since even in this relatively simple machine the qualitative behavior is well recovered at least up to $N\le 8$ for up to 5 ring-exchange times, we expect that using a combination of other error-mitigation techniques^29^, in particular zero-noise extrapolation, should significantly improve the results for larger lattices. This lies however beyond the scope of the present work.

## Methods

### Quantum simulation

We have presented results for the dynamics of RK ladders with 4, 6, and 8 plaquettes, which are described, respectively, by 3, 5 and 8 effective basis states, and which may be then evaluated using, respectively, 2, 3, and 3 qubits (note that the simplest case with just two plaquettes, which we do not discuss, may be simulated with a single qubit). In order to simulate the dynamics with a quantum computer, we first decompose the effective Hamiltonian $\hat{\mathcal {H}}_{eff}$ into a string of products of Pauli matrices *X* , *Y* and *Z*, and the identity *I*^36^. Table 1 provides the number of Pauli terms in the decomposition for different number *N* of plaquettes in the RK ladder. For $N=6$, the 3-qubit evaluation of the effective Hamiltonian in terms of Pauli terms acquires the form (for $\lambda =1$):

15 $$\begin{aligned} \hat{\mathcal {H}}_{\text {eff}} & = 2.25~ III - 0.612 ~IIX + 0.75 ~IIZ - 0.35~ IXI \nonumber \\ & \quad - 0.5 ~IXX + 0.35~ IXZ - 0.5 ~IYY + 1.0~ IZI \nonumber \\ & \quad - 0.612 ~IZ + 0.5 ~IZZ - 0.43~ XXI - 0.43 ~XXZ \nonumber \\ & \quad - 0.43~ YYI - 0.43 ~YYZ + 1.5 ~ZII - 0.612~ ZIX \nonumber \\ & \quad - 0.35 ~ZXI - 0.5 ~ZXX + 0.35 ~ZXZ - 0.5 ~ZYY \nonumber \\ & \quad + 0.25 ~ZZI - 0.612~ ZZX - 0.25~ ZZZ. \end{aligned}$$

**Fig. 4:**
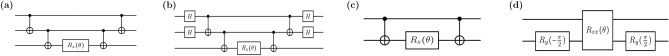
Standard circuit implementations of (**a**) $e^{-iZZZ \delta t}$ and (**b**) $e^{-iXXZ \delta t}$, where $\theta = 2\delta t$; (**c**) $R_{ZZ}(\theta )$ and (*d*) scaled $R_{ZZ}(\theta )$ based on cross-resonance formalism.

Expressing this linear superposition as $\hat{\mathcal {H}}_{\text {eff}}= \sum _k \hat{h}_k$, we may then evaluate the evolution operator using first order Trotter-Suzuki approximation^37,38^

16 $$\begin{aligned} e^{-i\hat{\mathcal {H}}_{\text {eff}}t} \approx \left( \prod _k e^{-i\hat{h}_k\delta t} \right) ^n \end{aligned}$$

where $t=n \delta t$, *n* is number of Trotter steps and $\delta t$ is the Trotter time step. In a standard quantum circuit implementation, each evolution operator $e^{-i\hat{h}_k \delta t}$ is decomposed into single qubit rotations and CNOT gates^39,40^, as illustrated in the examples of Fig. 4a,b.

### Scaled quantum gates

In NISQ devices it is crucial to reduce as much as possible the number and duration of two-qubit operations, which constitute the main source of errors. Alternative implementations have been hence recently proposed^41–43^, in which the use of scaled quantum gates allows to decrease the number of two-qubit operations, leading to a significant error reduction in the implementation.

In IBM-Q machines, the CNOT operation is natively realized by the two-qubit rotation $R_{ZX}(\pi /2)$ where Pauli *Z* and *X* gates act on driven control and target qubits, respectively, implemented by echoed cross-resonance (CR) pulses^44^. A scaled $R_{ZX}(\theta )$ can be implemented with CR pulses, where the rotation angle $\theta$ depends on the pulse area. This scaled $R_{ZX}(\theta )$ is then used to implement a scaled $R_{ZZ}(\theta )$ as shown in Fig. 4c,d. Note that $R_{ZZ}(\theta )$ is central in the implementation of any interaction term of the effective Hamiltonian. Since errors mostly arise due to the CR pulses, the use of scaled $R_{ZZ}(\theta )$ to implement all the terms of the evolution operator reduces the overall error in the circuit by decreasing both the overall duration of the pulse schedule and the number of native CNOT gates in the circuit. Table 1 provides the number of native CNOT gates in the basis gate set implementation, and the number of remaining CNOT gates in the scaled gate implementation for *N* plaquettes in one circuit repetition.

**Table 1:** For a given number of plaquettes, the table presents the number of Pauli terms obtained when decomposing the Hamiltonian into a sum of products of Pauli matrices, as in Eq. (15), the number of native (IBM-Q) CNOT gates per Trotter step in the standard circuit implementation of unitary evolution of the Hamiltonian, and the number of remaining native (IBM Q) CNOT gates per Trotter step in the scaled-gate circuit implementation of the unitary evolution operator.

Number of plaquettes	No. Pauli terms	No. CNOT (native)	No. CNOT (scaled)
4	7	6	0
6	23	48	14
8	26	64	20

Supplementary material presents a detailed description of the generation of scaled gates and of the analysis of the average gate fidelity for the scaled implementation of $R_{ZX}(\theta )$ against the standard CNOT implementation.

## Electronic supplementary material

Below is the link to the electronic supplementary material.

Supplementary Material 1

## Data Availability

The source code is publicly available in this (Githubrepository). All data underlying the results is also included in the Github repository.
