# Supplementary material for: Simulation of a Rohksar–Kivelson ladder on a NISQ device
Source: Sci Rep. 2024 Nov 26;14:29276. doi: 10.1038/s41598-024-79480-2 (PMC11589766; doi:10.1038/s41598-024-79480-2)
Supplement: Supplementary file 1 — Supplementary Material 1 [file 41598_2024_79480_MOESM1_ESM.pdf]

Supplementary material

Generation of scaled gates

In this section, we discuss the generation of scaled $R_{ZZ}(\theta)$ gates from a scaled $R_{ZX}(\theta)$ gate using Qiskit Pulse[1,2]. Since a longer pulse duration results in more error, $R_{ZZ}(\theta)$ can be scaled to a lesser pulse schedule duration and hence lesser two-qubit error than the CNOT based implementation of R_{ZZ}. The scaled $R_{ZX}(\theta)$ is implemented by modifying the amplitude and duration of the $R_{ZX}(\pi/2)$ gate , which generates the fundamental entangling operation for a CNOT gate, as outlined in Ref.[3,4]. The $R_{ZX}(\pi/2)$ is realized by echoed cross-resonance CR($\pm\pi/4$) pulses and an X-echoed π-pulse applied on the control qubit. The X-echoed π-pulse serves to minimize the effect of ZI and IX interaction terms[1,5,6]. Effects of other terms like ZZ and IY can be suppressed by applying certain rotary pulses[7]. A perfect $R_{ZX}(\pi/2)$ gives rise to high fidelity entangling operation. The CR pulse has a Gaussian flat-top waveform, and has as attributes its flat-top width w, its amplitude a, its duration d, and the number of standard deviations of Gaussian tails contained in the pulse n_σ. The area A under the Gaussian pulse is then given by

$$A = |a|w + |a|\sigma\sqrt{2\pi}\,\mathrm{erf}(n_\sigma). \tag{1}$$

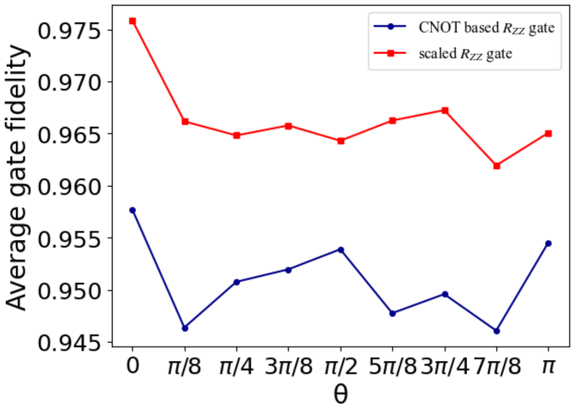

Figure 1. Comparison of the average gate fidelity for CNOT-based and scaled $R_{ZX}(\theta)$-based implementations of R_{ZZ} for various angles, calculated for the IBM-Q Lagos device.

To scale the pulse, depending on an arbitrary angle θ, the area under the CR Gaussian pulse is modified relative to the area $A(\pi/2)$ of the CR pulse for $R_{ZX}(\pi/2)$ such that the area under the modified pulse $A(\theta)$ is given by

$$A(\theta) = \frac{\theta}{\pi/2} A(\pi/2). \tag{2}$$

The modification in the pulse is achieved by either changing the width of the pulse or the amplitude, depending upon the initial parameters of the pulse. When $A(\theta) > |a(\pi/2)|\sigma\sqrt{2\pi}\,\mathrm{erf}(n_\sigma)$, the pulse width is modified as

$$w(\theta) = \frac{a(\theta)}{|a(\pi/2)|} - \sigma\sqrt{2\pi}\,\mathrm{erf}(n_\sigma). \tag{3}$$

When $A(\theta) < |a(\pi/2)|\sigma\sqrt{2\pi}\,\mathrm{erf}(n_\sigma)$, the flat-top width becomes 0 and we scale the amplitude as

$$|a(\theta)| = \frac{A(\theta)}{\sigma\sqrt{2\pi}\,\mathrm{erf}(n_\sigma)}. \tag{4}$$

We perform a simple Quantum Process Tomography experiment using qiskit-experiments library[8] to calculate the average gate fidelity for both the implementations of R_{ZZ} for various angles. We find that the scaled-R_{ZZ} has better fidelity than the CNOT-based implementation for all angles as shown in Fig. 1.

Mapping for ladders with 4 and 8 plaquettes

Using the same notation as in the main text, the effective basis of states for the case of RK-ladders with $N = 4$ plaquettes contains only 3 states:

$$\begin{aligned}
|\psi_0\rangle &= |0\rangle \\
|\psi_1\rangle &= \frac{1}{\sqrt{4}}(|1\rangle + |2\rangle + |3\rangle + |4\rangle) \\
|\psi_2\rangle &= \frac{1}{\sqrt{2}}(|13\rangle + |24\rangle)
\end{aligned} \tag{5}$$

Using these basis states, we can rewrite the effective Hamiltonian as

$$\hat{\mathscr{H}}_{eff} = \begin{pmatrix} 4\lambda & -2J & 0 \\ -2J & 2\lambda & -\sqrt{2}J \\ 0 & -\sqrt{2}J & 2\lambda \end{pmatrix} \tag{6}$$

We map the Hamiltonian for 4 plaquettes to an effective 3 states which can be simulated using 2 qubits.

For the case of $N = 8$ plaquettes, the effective basis has 8 states:

We define the basis states as

$$\begin{aligned}
|\psi_0\rangle &= |0\rangle \\
|\psi_1\rangle &= \frac{1}{\sqrt{8}}(|1\rangle + |2\rangle + |3\rangle + |4\rangle + |5\rangle + |6\rangle + |7\rangle + |8\rangle) \\
|\psi_2\rangle &= \frac{1}{\sqrt{8}}(|13\rangle + |17\rangle + |24\rangle + |28\rangle + |35\rangle + |46\rangle + |57\rangle + |68\rangle) \\
|\bar{\psi}_2\rangle &= \frac{1}{\sqrt{8}}(|14\rangle + |16\rangle + |25\rangle + |27\rangle + |36\rangle + |38\rangle + |47\rangle + |58\rangle) \\
|\bar{\psi}_2'\rangle &= \frac{1}{\sqrt{4}}(|15\rangle + |26\rangle + |37\rangle + |48\rangle) \\
|\psi_3\rangle &= \frac{1}{\sqrt{8}}(|135\rangle + |137\rangle + |247\rangle + |248\rangle + |357\rangle + |468\rangle + |157\rangle + |268\rangle) \\
|\bar{\psi}_3\rangle &= \frac{1}{\sqrt{8}}(|147\rangle + |146\rangle + |258\rangle + |257\rangle + |136\rangle + |368\rangle + |247\rangle + |358\rangle) \\
|\psi_4\rangle &= \frac{1}{\sqrt{2}}(|1357\rangle + |2468\rangle),
\end{aligned} \tag{7}$$

Using these basis states, we can write the Hamiltonian in the form:

$$\hat{\mathscr{H}}_{eff} = \begin{pmatrix}
8\lambda & -\sqrt{8}J & 0 & 0 & 0 & 0 & 0 & 0 \\
-\sqrt{8}J & 6\lambda & -2J & -2J & -\sqrt{2}J & 0 & 0 & 0 \\
0 & -2J & 5\lambda & 0 & 0 & -2J & -1J & 0 \\
0 & -2J & 0 & 4\lambda & 0 & 0 & -2J & 0 \\
0 & -\sqrt{2}J & 0 & 0 & 4\lambda & -\sqrt{2}J & 0 & 0 \\
0 & 0 & -2J & 0 & -\sqrt{2}J & 4\lambda & 0 & -\sqrt{4}J \\
0 & 0 & -1J & -2J & 0 & 0 & 3\lambda & 0 \\
0 & 0 & 0 & 0 & 0 & -\sqrt{4}J & 0 & 4\lambda
\end{pmatrix} \tag{8}$$

The corresponding dynamics can be then simulated using only three qubits.

References

1. Alexander, T. *et al.* Qiskit pulse: programming quantum computers through the cloud with pulses. *Quantum Sci. Technol.* **5**, 044006, DOI: 10.1088/2058-9565/aba404 (2020).
2. Aleksandrowicz, G. *et al.* Qiskit: An Open-source Framework for Quantum Computing, DOI: 10.5281/zenodo.2562111 (2019).
3. Stenger, J. P. T., Bronn, N. T., Egger, D. J. & Pekker, D. Simulating the dynamics of braiding of majorana zero modes using an ibm quantum computer. *Phys. Rev. Res.* **3**, 033171, DOI: 10.1103/PhysRevResearch.3.033171 (2021).
4. Chen, I.-C., Burdick, B., Yao, Y., Orth, P. P. & Iadecola, T. Error-mitigated simulation of quantum many-body scars on quantum computers with pulse-level control. *Phys. Rev. Res.* **4**, 043027, DOI: 10.1103/PhysRevResearch.4.043027 (2022).
5. Sheldon, S., Magesan, E., Chow, J. M. & Gambetta, J. M. Procedure for systematically tuning up cross-talk in the cross-resonance gate. *Phys. Rev. A* **93**, 060302, DOI: 10.1103/PhysRevA.93.060302 (2016).
6. Magesan, E. & Gambetta, J. M. Effective hamiltonian models of the cross-resonance gate. *Phys. Rev. A* **101**, 052308, DOI: 10.1103/PhysRevA.101.052308 (2020).
7. Sundaresan, N. *et al.* Reducing unitary and spectator errors in cross resonance with optimized rotary echoes. *PRX Quantum* **1**, 020318, DOI: 10.1103/PRXQuantum.1.020318 (2020).
8. Kanazawa, N. *et al.* Qiskit experiments: A python package to characterize and calibrate quantum computers. *J. Open Source Softw.* **8**, 5329, DOI: 10.21105/joss.05329 (2023).
